# Feasibility of Using ^18^F-FDG PET/CT Radiomics and Machine Learning to Detect Drug-Induced Interstitial Lung Disease

**DOI:** 10.3390/diagnostics14222531

**Published:** 2024-11-12

**Authors:** Charlotte L. C. Smith, Gerben J. C. Zwezerijnen, Sanne E. Wiegers, Yvonne W. S. Jauw, Pieternella J. Lugtenburg, Josée M. Zijlstra, Maqsood Yaqub, Ronald Boellaard

**Affiliations:** 1 Department of Radiology and Nuclear Medicine, Amsterdam UMC Location Vrije Universiteit Amsterdam, Boelelaan 1117, 1081 HV Amsterdam, The Netherlands; 2 Imaging and Biomarkers, Cancer Center Amsterdam, 1081 HV Amsterdam, The Netherlands; 3 Department of Hematology, Amsterdam UMC Location Vrije Universiteit Amsterdam, Boelelaan 1117, 1081 HV Amsterdam, The Netherlands; 4 Department of Hematology, Erasmus MC Cancer Institute, University Medical Center Rotterdam, 3015 CN Rotterdam, The Netherlands

**Keywords:** drug-induced interstitial lung disease, bleomycin, ^18^F-FDG PET/CT, machine learning and radiomics

## Abstract

Background: Bleomycin is an oncolytic and antibiotic agent used to treat various human cancers because of its antitumor activity. Unfortunately, up to 46% of the patients treated with bleomycin develop drug-induced interstitial lung disease (DIILD) and potentially life-threatening interstitial pulmonary fibrosis. Tools and biomarkers for predicting and detecting DIILD are limited. Therefore, we aimed to evaluate the feasibility of ^18^F-FDG PET/CT, PET radiomics, and machine learning in distinguishing DIILD in an explorative pilot study. Methods: Eighteen Hodgkin’s lymphoma (HL) patients, of whom 10 developed DIILD after treatment with bleomycin, were retrospectively included. Five diffuse large B-cell lymphoma (DLBCL) patients were included as a control group since they were not treated with bleomycin. All patients underwent ^18^F-FDG PET/CT scans before (baseline) and during treatment (interim). Structural changes were assessed by changes in Hounsfield Units (HUs). The ^18^F-FDG PET scans were used to assess metabolic changes by examining the feasibility of 504 radiomics features, including the mean activity of the lungs (SUVmean). A Random Forest (RF) classifier evaluated the identification and prediction of DIILD based on PET radiomics features. Results: HL patients who developed DIILD showed a significant increase in standard SUV metrics (SUVmean; *p* = 0.012, median increase 37.4%), and in some regional PET radiomics features (texture strength; *p* = 0.009, median increase 101.6% and zone distance entropy; *p* = 0.019, median increase 18.5%), while this was not found in HL patients who did not develop DIILD and DLBCL patients. The RF classifier correctly identified DIILD in 72.2% of the patients and predicted the development of DIILD correctly in 50% of the patients. There were no significant differences in HUs over time within all three patient groups. Conclusions: Our explorative longitudinal pilot study suggests that certain regional ^18^F-FDG PET radiomics features can effectively identify DIILD in HL patients treated with bleomycin, as significant longitudinal increases were observed in SUVmean, texture strength, and zone distance entropy after the development of DIILD. The metabolic activity of these features did not significantly increase over time in DLBCL patients and HL patients who did not develop DIILD. This indicates that ^18^F-FDG PET radiomics, with and without machine learning, might serve as potential biomarkers for detecting DIILD.

## 1. Introduction

Bleomycin was discovered by Umezawa et al. [1] as an antibiotic agent with antitumor activity. Nowadays, bleomycin treats various human cancers such as germ cell tumors, squamous cell carcinoma, testicular cancer, and Hodgkin’s lymphoma (HL) [2,3]. Unfortunately, up to 46% of the patients treated with bleomycin can develop drug-induced interstitial lung disease (DIILD) due to the limited hydrolysis of bleomycin in the lungs [4,5]. Additionally, life-threatening interstitial pulmonary fibrosis occurs in approximately 1% to 2% of patients [4]. Although nintedanib and pirfenidone can effectively decelerate the progression of interstitial pulmonary fibrosis, the disease remains progressive and fatal [6,7].

Parenchymal abnormalities are typically detected with high-resolution computed tomography (HRCT) scans due to their high sensitivity [8]. However, HRCT scans expose patients to high radioactive dosages, and it remains unclear whether structural changes can predict DIILD in patients treated with bleomycin. The development of DIILD is associated with several risk factors, such as age, cumulative bleomycin dose, impaired renal function, smoking, and pulmonary radiation [4,9], yet clear biomarkers are still limited. Several case studies using ^18^F-fluoro-deoxy-glucose (^18^F-FDG) positron emission tomography–computed tomography (PET/CT) imaging in HL and seminoma patients did not find structural differences in patients with DIILD [10,11,12,13,14]. In contrast, a review by Exarchos et al. [15] highlighted the potential for combining CT scans with machine learning in classifying and predicting interstitial lung disease (ILD). Additionally, the aforementioned case studies indicated elevated metabolic activity during DIILD, suggesting a potential alteration in pulmonary metabolic processes secondary to DIILD [10,11,12,13,14]. Notably, the study by Beyhan Sagmen et al. [16] observed increased ^18^F-FDG pulmonary uptake in HL patients after the initiation of doxorubicin, bleomycin, vinblastine, and dacarbazine (ABVD) chemotherapy, indicative of subsequent development of DIILD compared to pre-treatment levels. This was not found in HL patients who did not develop DIILD after treatment with ABVD chemotherapy [16]. These promising results suggest that metabolic alterations in the lungs could serve as biomarkers for DIILD. However, it remains unclear whether these results apply solely to conventional standardized uptake value (SUV) metrics or if they also apply to more advanced PET features, including inter- and intravoxel-based features.

Inter- and intravoxel-based features of regional ^18^F-FDG PET uptake, commonly referred to as radiomics, are gaining importance in oncology [17,18]. They are extensively studied for prognostic assessments and predicting treatment responses, effectively forming a comprehensive set of biomarkers [17,18]. Exploring the feasibility of radiomics features through a longitudinal pilot study could enhance DIILD understanding, as these features can reveal biological characteristics and capture more repeatable and reproducible quantitative image information [17,18]. This may enable early detection and disease onset prediction by identifying advanced PET characteristics beyond the conventional SUV metrics. Additionally, integrating PET radiomics features with artificial intelligence (AI) could be valuable, since previous studies have shown that combining radiomics with machine learning methods, such as a Random Forest (RF), improves the robustness of the statistical analysis [15,19].

In this study, we aimed to analyze, through an exploratory longitudinal pilot study, the feasibility of standard ^18^F-FDG PET/CT features and advanced ^18^F-FDG PET radiomics features combined with an RF classifier to classify DIILD in HL patients treated with bleomycin. To achieve this, we included HL patients who developed DIILD and HL patients who did not develop DIILD after several cycles of chemotherapy, including bleomycin as an antitumor agent. We also included diffuse large B-cell lymphoma (DLBCL) patients as a control group since they did not receive bleomycin during treatment.

## 2. Materials and Methods

### 2.1. Patient Datasets

Patients were retrospectively included from 2009 to 2021. We included ten HL patients who developed DIILD and eight HL patients who did not develop DIILD after several treatment cycles with bleomycin, etoposide, doxorubicin, cyclophosphamide, vincristine, procarbazine, and prednisolone (BEACCOPP) or ABVD. Additionally, seven DLBCL patients were included as a control group since they were not treated with bleomycin as an oncolytic agent. Clinical data and the ^18^F-FDG PET/CT scans of the HL patients were collected at different hospitals (*n* = 8) in the Netherlands. The ^18^F-FDG PET/CT scans and clinical data of the DLBCL patients were collated and harmonized by the HOVON-84 study [20]. All ^18^F-FDG PET/CT scans were collected and harmonized according to the European Association of Nuclear Medicine Research Ltd. (EARL) guidelines for multicenter PET images. The VU Medical Centre ethics review board waived the use of anonymized clinical data (IRB ID: 2022.072).

Patients were retrospectively included when they received at least two longitudinal ^18^F-FDG PET/CT scans. The first ^18^F-FDG PET/CT scan needed to be before treatment (baseline), and the other scan must have been performed during treatment (interim). Furthermore, patient data such as patient weight, age, and gender needed to be available. For HL patients, treatment with either BEACCOPP or ABVD was required. Additionally, the development of DILLD during interim PET imaging needed to be documented, as assessed by HRCT scan or clinical data such as respiratory information. All scans were subject to strict quality control (QC) requirements. Scans passed QC when (1) whole-body ^18^F-FDG PET/CT scans were complete, (2) essential Digital Imaging and Communications in Medicine (DICOM) information was available, (3) plasma glucose levels were within the ranges suggested by the EARL guidelines [18], and (4) the liver SUVmean was within the suggested range (1.3–3.0) measured using a volume of interest (VOI) with a diameter of 3 cm placed in the (unaffected) right upper lobe of the liver [18]. Patients were excluded if their scans did not meet QC requirements or if they experienced active infectious lung injury unrelated to DIILD. Moreover, DLBCL patients treated with bleomycin between baseline and interim imaging were excluded as well.

### 2.2. ^18^F-FDG PET/CT Analysis

All scans were performed and reconstructed according to the EARL guidelines to obtain images adhering to the European guidelines for multicenter PET image harmonization and quantification [21]. In short, all patients fasted for 4–6 h before tracer administration, had plasma glucose levels < 7.0 mmol/L, and received 3.0 MBq/kg radioactive tracer through an intravenous bolus injection. Low-dose CT (LDCT) scans were used to correct the PET images for attenuation.

**Figure 1:**
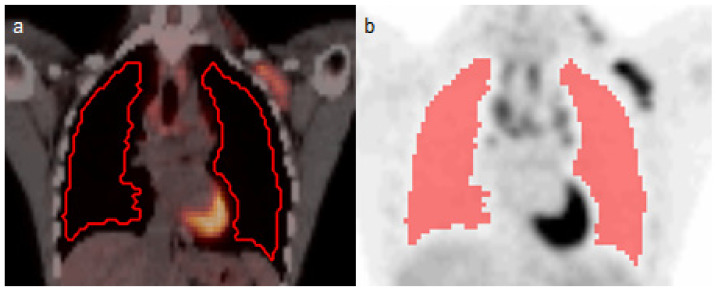
The lung volume of interest (VOI) applied to the (**a**) CT fused with the PET and (**b**) PET image.

The lungs were segmented with the in-house-build semi-automated analysis software tool ACCURATE (developed in IDL version 8.4 (Harris Geospatial Solutions, Bloomfield, NJ, USA)) [22]. The lungs were initially automatically segmented based on the LDCT images using a convolutional neural network (CNN). All segmentations were eroded to minimize the overlap of the VOI with other organs or blood vessels when applying the VOI to the PET images. The VOIs were then applied to the PET images and visually inspected by a (+5-year experienced) nuclear medicine physician. Manual adjustments were made if tumor tissue or other organs were included in the VOI. Figure 1 illustrates the lung VOI after adjustment in the LDCT fused with the PET image (Figure 1a) and in the PET image (Figure 1b).

The delineations from the LDCT scans were used to assess differences in Hounsfield Units (HUs). The mean activity from the ^18^F-FDG PET delineations was extracted to assess metabolic changes by examining the mean activity in the VOI normalized by body weight (SUVmean).

### 2.3. PET Radiomics

Image biomarker standardization initiative (IBSI)-compliant PET radiomics features were extracted for all ^18^F-FDG PET images using the RaCaT software (version 1.27) [23]. To avoid multicollinearity, we first assessed the PET radiomics features for strong correlations (r < −0.7 or r > 0.7) [24] with SUVmean, volume, or other features within HL patients who developed DIILD. Features strongly correlating with either SUVmean or volume were excluded from further analysis. The remaining features were then tested for strong correlations (r < −0.7 or r > 0.7) with each other. For the strong correlating features, those with the strongest association with all other features were excluded from further analyses (i.e., pairwise elimination). The final set of features that did not show a strong correlation with SUVmean, volume, or each other was used to assess the feasibility of radiomics in identifying DIILD in patients treated with bleomycin. These features along with SUVmean and volume were employed in an RF classifier, within the ‘RandomForest’ R package (version 4.7.1.1), to explore the potential of PET radiomics combined with machine learning for classifying and predicting DIILD. We analyzed the interim scans of HL patients who developed DIILD versus those who did not and evaluated the accuracy with which the features classified DIILD. The baseline scans examined how well the features could predict DIILD.

The mean decrease in the Gini index assessed the importance of the features. The RF classifiers were optimized for the number of trees (ntree) and the number of input variables (mtry), with cross-validation performed using the Leave-One-Out Cross-Validation (LOOCV) method in the ‘caret’ R package to provide an unbiased estimate of model performance. From the best model, the out-of-bag (OOB) predictions of the probability of developing DIILD were obtained for each patient. Subsequently, the Euclidean distance matrix based on the OOB prediction probabilities, which represents the dissimilarity between patients based on their probability of developing DIILD, was calculated. The dissimilarity between patients was visualized in a reduced dimensional space (2D) with classic multidimensional scaling (MDS). Therefore, the Euclidean distance matrix was subjected to double centering to obtain the eigendecomposition. The top two dimensions (MDS1 and MDS2), corresponding to the two largest eigenvalues, were extracted to create a 2D representation of the data. This reduction aims to preserve the pairwise distances between patients as accurately as possible.

### 2.4. Statistical Analysis

Statistical analyses and data visualization were performed in R (version 4.2.2). Longitudinal differences in HUs and SUVmean (between baseline and interim scans) were described using the median and interquartile ranges (IQRs) and represented with line plots. Within the three groups, differences in HUs and SUVmean over time were represented with Tukey’s boxplots. Statistical differences over time within the groups were tested for significance (*p* ≤ 0.050) with a Wilcoxon signed-rank test. The Wilcoxon signed-rank test was used to test the remaining PET radiomics features for significance (*p* ≤ 0.050) between baseline and interim values within HL patients who developed DIILD. The features that showed significant longitudinal differences between baseline and interim scans in HL patients who developed DIILD were also evaluated for significant differences between baseline and interim values in HL patients who did not develop DIILD and in DLBCL patients. Additionally, the remaining PET radiomics features together with SUVmean and volume were used in an RF classifier to examine the feasibility of PET radiomics features in classifying and predicting DIILD in HL patients. All tests were performed non-parametrically due to the small sample size.

## 3. Results

### 3.1. Patient Demographics

Initially, 18 HL patients and 7 DLBCL patients were included. After visual inspection, two DLBCL patients were excluded from further analysis due to active infectious lung injury during the interim ^18^F-FDG PET/CT scan. This resulted in 18 HL patients (44.4% female) and 5 DLBCL patients (40.0% female). Ten (55.6%) HL patients developed DIILD (30.0% female) during treatment. There were no significant group differences in age and gender (*p* > 0.050). Patient demographics are summarized in Table 1.

### 3.2. CT Analysis Using Hounsfield Units

**Figure 2:**
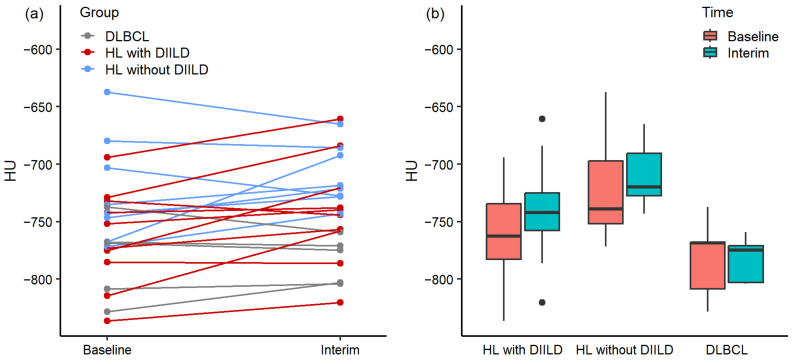
Differences in Hounsfield Units (HUs) between baseline and interim LDCT scans for Hodgkin’s lymphoma (HL) patients who developed drug-induced interstitial lung disease (DIILD), HL patients who did not develop DIILD, and patients with diffuse large B-cell lymphoma (DLBCL) (**a**) for each patient individually and (**b**) for each patient group. The central line of the box represents the median, and the edges are the 25th and 75th percentiles. The extreme data points which are not considered outliers are illustrated with the black dots.

Differences in HUs of the lungs over time are illustrated in Figure 2a,b. Individual differences over time are illustrated in Figure 2a, and group differences over time are illustrated in Figure 2b. Within every group, HUs decreased, increased, or stayed stable over time (Figure 2a). There were no significant differences in HUs between baseline and interim LDCT scans for all three patient groups (*p* > 0.050). HL patients who developed DIILD (median −762.53 and IQR 48.18 at baseline, median −741.96 and IQR 32.60 at interim) showed a median HU decrease of 2.7% between baseline and interim LDCT scans. HL patients without DIILD (median −738.94 and IQR 54.73 at baseline, median −719.71 and IQR 36.92 at interim) had a median HU decrease of 2.6%, and DLBCL patients (median −768.40 and IQR 40.91 at baseline, median −774.67 and IQR 32.07 at baseline) showed a median HU increase of 0.8% between the baseline and interim scans.

### 3.3. ^18^F-FDG PET Standardized Uptake Value

**Figure 3:**
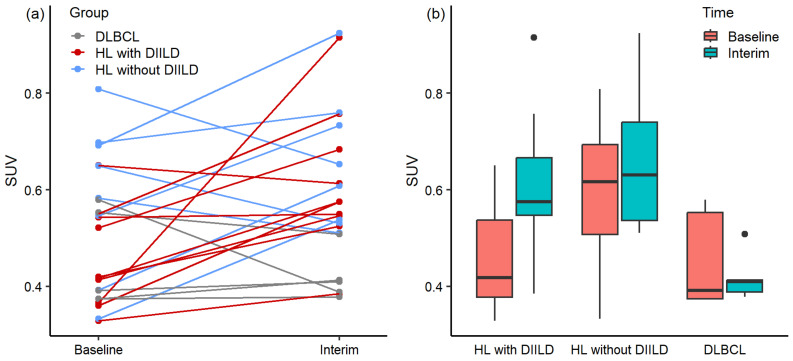
Differences in lung SUVmean between baseline and interim ^18^F-FDG PET scans for Hodgkin’s lymphoma (HL) patients who developed drug-induced interstitial lung disease (DIILD), HL patients who did not develop DIILD, and patients with diffuse large B-cell lymphoma (DLBCL) (**a**) for each patient individually and (**b**) for each patient group. The central line of the box represents the median, and the edges are the 25th and 75th percentiles. The extreme data points which are not considered outliers are illustrated with the black dots.

Differences in lung SUVmean are illustrated in Figure 3a,b. Figure 3a illustrates individual differences, and group differences over time are illustrated in Figure 3b. The SUVmean increased over time in almost all HL patients who developed DIILD (Figure 3a). This was not observed in HL patients who did not develop DIILD and in DLBCL patients since the SUVmean of these patients increased, decreased, or stayed stable over time. HL patients who developed DIILD showed a significantly higher lung SUVmean at the interim ^18^F-FDG PET scan compared with baseline ^18^F-FDG PET scans (*p* = 0.012, median 0.42 and IQR 0.17 at baseline, median 0.58 and IQR 0.12 at interim). There were no significant differences in lung SUVmean between baseline and interim ^18^F-FDG PET scans for HL patients who did not develop DIILD (*p* > 0.050, median 0.62 and IQR 0.19 at baseline, median 0.63 and IQR 0.20 at interim) and DLBCL patients (*p* > 0.050, median 0.40 and IQR 0.18 at baseline, median 0.41 and IQR 0.02 at interim). The median increase in the lung SUVmean between baseline and interim ^18^F-FDG PET scans was 37.4% for HL patients who developed DIILD, 2.4% for the HL patients who did not develop DIILD, and 4.6% for DLBCL patients.

### 3.4. ^18^F-FDG PET Radiomics and Machine Learning Analysis

**Figure 4:**
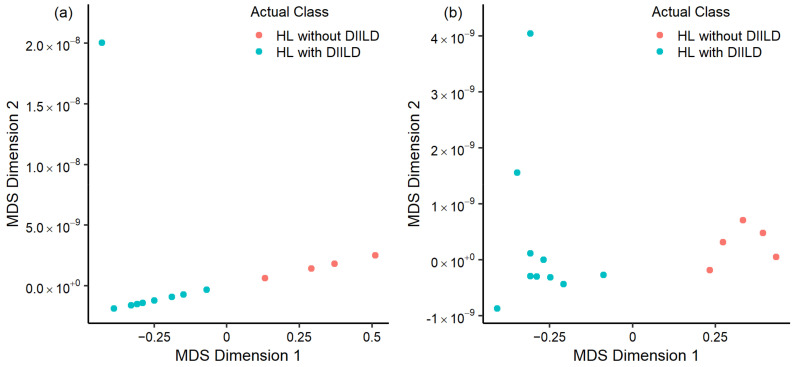
Multidimensional scaling (MDS) plot derived from the Random Forest out-of-bag predictions from the (**a**) interim and (**b**) baseline samples of Hodgkin’s lymphoma (HL) patients who developed drug-induced interstitial lung disease (DIILD) and HL patients who did not develop DIILD. The data are represented in two dimensions and capture the primary source of variance in the predicted probabilities of developing DIILD. Each data point corresponds to an individual patient, and the color indicates the actual occurrence of DIILD.

Eight radiomics features (90th intensity percentile, D max bulk, elongation, coefficient of variance, coarseness, small zone emphasis, texture strength, and zone distance entropy) did not correlate significantly with SUVmean, volume, or each other within HL who developed DIILD. Even though all features showed an absolute correlation smaller than 0.7, the 90th intensity percentile feature was still excluded to avoid multicollinearity due to its high correlations with texture strength (r = 0.692) and SUVmean (r = 0.647). The seven remaining features together with SUVmean and volume were used in the RF classifier. After model optimization and validation with the LOOCV method, the best RF classifier based on interim values of HL patients who developed DIILD and HL patients who did not develop DIILD contained 50 trees with seven input variables. The accuracy of the best model was 72.22% (OOB error rate: 27.78%) with texture strength as the most important feature. Feature importance based on the mean decrease in the Gini index is summarized in Table 2. The predicted probabilities of the development of DIILD are illustrated in Figure 4a with an MDS plot. The data points in the plot are colored based on the actual occurrence of DIILD and patients with similar predictions are clustered together. The MDS plot shows that there is a clear clustering between HL patients who developed DIILD and patients who did not. The baseline values of HL patients who will develop DIILD and HL patients who will not were used to predict the development of DIILD before treatment with bleomycin. After cross-validation, the best RF classifier based on baseline values contained 50 trees with two input variables. The accuracy of the best model was 50% (OOB error rate: 50%) with zone distance entropy as the most important feature. Feature importance based on the mean decrease in the Gini index is summarized in Table 2. The predicted probabilities of the development of DIILD based on baseline values are illustrated in Figure 4b with an MDS plot. The data points in the plot are colored based on the actual development of DIILD, and patients with similar predictions are clustered together. Figure 4b shows a clear clustering between HL patients who will develop DIILD and HL patients who will not after several cycles of BEACOPP or ABVD.

**Figure 5:**
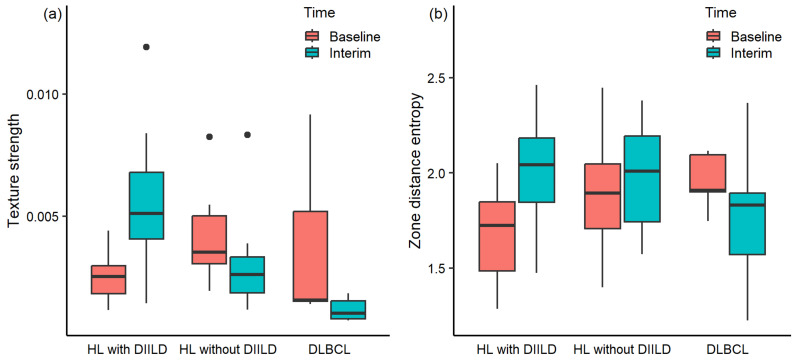
(**a**) Texture strength and (**b**) zone distance entropy in the lungs at baseline and interim ^18^F-FDG PET scans for Hodgkin’s lymphoma (HL) patients who developed drug-induced interstitial lung disease (DIILD), HL patients who did not develop DIILD, and patients with diffuse large B-cell lymphoma (DLBCL). The central line of the box represents the median, and the edges are the 25th and 75th percentiles. The extreme data points which are not considered outliers are illustrated with the black dots.

After the seven features were tested for significance between baseline and interim values within HL patients who developed DIILD, two features (texture strength and zone distance entropy) exhibited a significant longitudinal increase (*p* = 0.009 and *p* = 0.019, respectively). The median increase over time was 101.6% (median 0.0025 and IQR 0.0011 at baseline, median 0.0051 and IQR 0.0027 at interim) for the texture strength feature and 18.5% for zone distance entropy (median 1.72 and IQR 0.36 at baseline, median 2.04 and IQR 0.34 at interim). There were no significant differences between baseline and interim PET scans for the texture strength and zone distance entropy features within HL patients who did not develop DIILD and DLBCL patients (*p* > 0.050). The median increase over time for texture strength was 26.0% (median 0.0035 and IQR 0.0020 at baseline, median 0.0026 and IQR 0.0015 at interim) in HL patients who did not develop DIILD and 34.2% (median 0.0016 and IQR 0.0037 at baseline, median 0.0010 and IQR 0.0007 at interim) for DLBCL patients. The median increase over time for zone distance entropy was 6.1% (median 1.89 and IQR 0.34 at baseline, median 2.01 and IQR 0.45 at interim) for HL patients who did not develop DIILD and 4.1% (median 1.91 and IQR 0.19 at baseline, median 1.83 and IQR 0.32 at interim) for DLBCL patients. The differences in texture strength and zone distance entropy over time for all three patient groups are illustrated in Figure 5a and Figure 5b, respectively.

## 4. Discussion

In this study, we aimed to examine the feasibility of standard ^18^F-FDG PET/CT metrics and ^18^F-FDG PET radiomics features, combined with machine learning, to potentially classify and predict bleomycin-induced ILD in HL patients. While our data did not reveal longitudinal differences in HUs in the lungs across all three patient groups, they identified that conventional SUV metrics, along with some advanced regional ^18^F-FDG PET radiomics features, may effectively indicate DIILD. Furthermore, machine learning approaches using advanced PET radiomics exhibit promise in classifying DIILD in HL patients treated with bleomycin.

Consistent with previous case studies [10,11,12,13,14], our findings indicated no structural differences over time based on LDCT scans. The inability to distinguish DIILD from LDCT scans reinforces the notion that LDCT scans cannot replace HDCT scans in detecting DIILD or even predicting the occurrence of DIILD when combining HDCT scans with AI [15]. Nevertheless, we did find increased metabolic lung activity, as reflected by a median increase of 37.4% in standard SUV metrics from baseline to interim ^18^F-FDG PET scans in HL patients who developed DIILD. In contrast, no significant metabolic changes were noted in HL patients who did not develop DIILD and DLBCL patients, aligning with previous studies [10,11,12,13,14,16]. Moreover, the regional ^18^F-FDG PET radiomics features texture strength and zone distance entropy may serve as effective metabolic classifiers to determine DIILD. Our preliminary results showed significant increases in texture strength (median increase of 101.6%) and zone distance entropy (median increase of 18.5%) over time in HL patients who developed DIILD, while these features remained stable in HL patients who did not develop DIILD and DLBCL patients. Differences in zone distance entropy were expected since this metric reflects the inhomogeneity of the lungs [17,25]. The inhomogeneity increases after the development of DIILD, as interim PET scans mostly showed inflammation in the lower part of the lungs during active DIILD. Texture strength, representing spatial changes in voxel intensity, belongs to second-order histograms with Neighborhood Grey-Tone Difference Matrices (NGTDMs) [17,25].

Our preliminary results suggest that combining advanced radiomics features with machine learning techniques can potentially classify DIILD in HL patients. The RF classifier based on interim values achieved a classification accuracy of 72%, and the MDS plot based on the Euclidian distance of the OOB prediction probabilities showed a clear distinction between patients who developed DIILD and those who did not. However, model accuracy dropped to 50% when predicting the development of DIILD in HL patients based on baseline PET radiomics values. Nonetheless, the MDS plot based on the baseline values showed a clear distinction between HL patients who will develop DIILD and those who will not, indicating that there might be a potential utility in predicting DIILD in HL patients treated with bleomycin. This is important since the use of baseline and interim ^18^F-FDG PET/CT scans in HL patients is becoming more common in clinical practice [26]. Utilizing these ^18^F-FDG PET/CT scans for the detection and prediction of DIILD might help in earlier detection.

To our knowledge, this is the first study to explore the potential of advanced PET radiomics features in classifying bleomycin-induced ILD. However, it should be emphasized that this is an explorative pilot study with preliminary results based on limited data. The small sample size limits our results. Several limitations were identified, including the small sample size, which restricted our ability to fully evaluate the feasibility of CT and PET features in classifying and predicting the occurrence of DIILD. A larger cohort is necessary for robust validation of our results. Additionally, the current RF classifier was trained exclusively on HL patients who either developed or did not develop DIILD during bleomycin treatment. As a result, external validation is warranted to assess the potential of the currently selected radiomics features combined with machine learning in classifying or predicting DIILD. The small sample size precluded statistical group comparisons. Although visual interpretation suggested possible group differences, it remains uncertain whether these differences are genuinely associated with an increased risk of developing drug-induced interstitial lung disease (DIILD) or are coincidental, potentially influenced by confounding factors such as age, smoking status, renal function, or the specific chemotherapy combination. Other limitations include variations in uptake times. Sometimes, the uptake times exceeded the recommendations of the EARL guidelines [21], which could influence the semi-quantitative analysis. Replicating the results with an uptake time between 55 and 75 min is therefore crucial [21]. Finally, we observed discrepancies in the intervals between baseline and interim scans among the groups since HL patients who developed DIILD had a longer mean interval compared to those who did not and the DLBCL cohort.

## 5. Conclusions

In conclusion, our exploratory pilot study assessed the feasibility of ^18^F-FDG PET/CT and ^18^F-FDG PET radiomics combined with machine learning to detect and predict bleomycin-induced ILD in HL patients. Our results underscore the potential for some regional ^18^F-FDG PET radiomics features, with and without machine learning, as valuable tools for identifying DIILD in HL patients treated with bleomycin. However, further research with a larger sample size, including an external validation cohort, is crucial to validate our findings and to establish ^18^F-FDG PET imaging as a reliable biomarker for DIILD detection and prediction. In addition, it is essential to extend this research to other patient groups receiving bleomycin beyond HL patients. While our study has limitations, it is noteworthy as the first longitudinal investigation into advanced PET radiomics as potential biomarkers for early DIILD detection. Early identification of DIILD is critical to help patients switch earlier to another antitumor agent to prevent the development of DIILD or life-threatening interstitial pulmonary fibrosis.

## Figures and Tables

**Table 1:** Patient demographics.

Characteristics	HL with DIILD (*n* = 10)	HL Without DIILD (*n* = 8)	DLBCL (*n* = 5)
Gender (F/M), number	3/7	5/3	2/3
Age (years), mean (range)	48.4 (23–69)	31.8 (18–46)	57.8 (30–68)
Injected dose (MBq), mean (SD)			
Baseline	190.8 (27.6)	274.2 (72.8)	233.3 (57.6)
Interim	195.4 (38.2)	268.3 (52.1)	243.6 (34.6)
Weight (kg), mean (SD)			
Baseline	66.7 (11.2)	78.8 (17.5)	73.8 (11.5)
Interim	66.3 (11.3)	78.9 (14.8)	70.6 (10.2)
Uptake time (min), mean (range)			
Baseline	70.8 (50–107)	66.3 (26–120)	68.7 (64–102)
Interim	71.9 (50–104)	68.7 (49–94)	65.2 (59–82)
Interval scans (week), mean (range)	22.6 (13–39)	13.8 (8–21)	10.2 (7–20)

Abbreviations: DIILD, drug-induced interstitial lung disease; DLBCL, diffuse large B-cell lymphoma; F, female; HL, Hodgkin’s lymphoma; kg, kilogram; M, male; MBq, mega Becquerel; min, minutes; *n*, number; SD, standard deviation.

**Table 2:** Feature importance based on the mean decrease in the Gini index.

Feature	Mean Decrease in Gini Index (Interim)	Mean Decrease in Gini Index (Baseline)
Zone distance entropy	0.287	1.373
Texture strength	2.703	1.351
SUVmean	0.412	1.049
Volume	0.797	0.971
D max Bulk	0.715	0.844
Elongation	0.566	0.730
Coarseness	0.822	0.726
Coefficient of variation	0.488	0.603
Small zone emphasis	1.341	0.590

## Data Availability

The datasets generated during and/or analyzed during the current study are available from the corresponding author upon reasonable request.
